# Evaluation of ViroTrack Sero Zika IgG/IgM, a New Rapid and Quantitative Zika Serological Diagnostic Assay

**DOI:** 10.3390/diagnostics10060372

**Published:** 2020-06-04

**Authors:** Tony Liao, Xiaole Wang, Marco Donolato, Eva Harris, Magelda Montoya Cruz, Angel Balmaseda, Robert Y.L. Wang

**Affiliations:** 1 Chang Gung Biotechnology Industry Master and PhD Program, Chang Gung University, Taoyuan 33302, Taiwan; tony.liao@blusense-diagnostics.com; 2 BluSense Diagnostics, Fruebjergvej 3, DK-2100 Copenhagen, Denmark; xiaole.wang@blusense-diagnostics.com (X.W.); marco@blusense-diagnostics.com (M.D.); 3 Division of Infectious Diseases and Vaccinology, School of Public Health, University of California, Berkeley, CA 94720, USA; eharris@berkeley.edu (E.H.); mageldamontoya@yahoo.es (M.M.C.); 4 National Virology Laboratory, National Center for Diagnosis and Reference, Ministry of Health, 107 Managua, Nicaragua; abalsameda@minsa.gob.ni; 5 Division of Pediatric Infectious Diseases, Department of Pediatrics, Chang Gung Memorial and Children’s Hospital, Linkuo 33305, Taiwan; 6 Department of Biomedical Sciences, College of Medicine, Chang Gung University, Taoyuan 33302, Taiwan

**Keywords:** diagnosis, dengue virus, Zika virus, immuno-magnetic assay, ViroTrack Sero Zika IgG/IgM

## Abstract

Dengue virus (DENV) and Zika virus (ZIKV) belong to the flavivirus genus and are antigenically closely related. They also share the same mosquito vector and can cause similar symptoms upon infection. However, DENV and ZIKV infections lead to different clinical sequelae and treatments; therefore, clinicians need rapid and accurate diagnostics capable of distinguishing between the two diseases. Methods: We employed the immuno-magnetic assay technology on a microfluidic cartridge (ViroTrack Sero Zika IgG/IgM) for diagnosis of ZIKV infection based on the aggregation of magnetic nanoparticles. We carried out three serological studies including samples from the Dominican Republic, USA, and Nicaragua, aimed at detecting ZIKV-specific IgG and IgM using the ViroTrack Sero Zika IgG/IgM test. Results: The seroconversion results were comparable with ZIKV IgG and IgM reactivity measured by the commercial ZIKV ELISA kit. The sensitivity and specificity for both ZIKV IgG and IgM tested by the ViroTrack Sero Zika IgG/IgM was approximately 98% and 93%, respectively. Conclusion: Serological detection of ZIKV infection by the new ViroTrack Sero Zika IgG/IgM test shows promising performance and limited cross-reactivity with DENV.

## 1. Introduction

Zika virus (ZIKV), an arthropod-borne virus (arbovirus) belonging to the flavivirus genus in the Flaviviridae family, became a global epidemic after its introduction into Brazil in 2014–2015 [1]. It spread rapidly throughout the Americas and caused severe clinical sequelae such as Guillain-Barré Syndrome (GBS) in adults, and microcephaly and other congenital defects in babies born to mothers infected during pregnancy in Oceania. There is still no cure for both severe diseases, raising the attention of the global audience. ZIKV is mainly transmitted by Aedes genus mosquitoes [2,3]. Other routes of transmission include occupational laboratory exposure [4], sexual intercourse [5,6], blood transfusion, and mother-to-child transmission [7,8,9,10]. ZIKV was first identified in Uganda in 1947 in monkeys [11]. No severe cases of Zika were reported until 2015, when the epidemic in Brazil caused hundreds of thousands of confirmed cases [12].

The incubation period of ZIKV is thought to be 3–14 days [13]. The typical symptoms are rash with or without fever, myalgia, and arthralgia, and can be similar to other flavivirus infections such as dengue virus (DENV) [14]. The symptoms are usually mild and last for only a few days. Since most cases don’t present with serious illness, this disease is easily ignored. However, ZIKV is associated with severe neurological diseases such as fetal microcephaly due to vertical transmission [15,16]. Defective brain development in the fetus causes symptoms in babies including seizures, developmental delays, intellectual disability and problems with movement and balance. Another disorder is GBS in adults [17], which is caused by the body’s immune system attacking myelin in the peripheral nervous system, resulting in improper signal transmission.

As with all flaviviruses, ZIKV is an enveloped, icosahedral virus. Its genome consists of positive-sense single-stranded RNA approximately 11 kb long, containing a single open reading frame encoding a polyprotein that is cleaved into three structural proteins—the capsid (C), membrane (prM/M), and envelope (E) proteins, and seven non-structural proteins—NS1, NS2A, NS2B, NS3, NS4A, NS4B, and NS5 [18,19]. The E protein is the major virion surface glycoprotein and is involved in various aspects of the viral cycle, mediating binding, endocytosis, and membrane fusion with the host cell. E protein is the main target for antibodies against the virus [20]. Among non-structural proteins, NS1 is a glycoprotein that has been reported to play different roles in virus replication, immune evasion, and pathogenesis [21,22,23]. Flavivirus NS1, as the only viral protein secreted from infected cells, has also been a useful biomarker because it can be detected in the blood of infected people at an early stage before the antibody response [24]. Thus, both E protein and NS1 play important roles in serological diagnosis of ZIKV infection.

Dengue virus, another flavivirus, shares the same mosquito vector as ZIKV. DENV has four serotypes and is widely spread throughout tropical and subtropical areas such as Asia and the Americas [25], where ZIKV is also prevalent. At the amino acid level, DENV and ZIKV E protein share 54–59% identity while NS1 proteins share 55% identity [26,27]. The co-circulation of DENV and ZIKV, and similarity in the amino acids of E protein and NS1, cause cross-reactivity problems in IgG and IgM serological diagnosis. The DENV infection could potentially be exacerbated due to cross-reactive antibodies from previous ZIKV infection through antibody-dependent enhancement (ADE) [28,29]. However, the cross-reactive neutralizing antibodies with high titers induced by ZIKV infection could possibly prevent the DENV infection [30,31,32]. Furthermore, the ZIKV and DENV infections also share similar symptoms at the initial stage, while ZIKV and DENV need different clinical case management, hence the need for new methods to distinguish both diseases.

Meanwhile, quick and accurate serological diagnosis of ZIKV infection is essential for patients needing special attention, such as pregnant women who need be monitored, particularly in the early stages of pregnancy. Therefore, there is an emergent need for rapid diagnostic tests (RDT) for ZIKV infection, especially in resource-limited regions. However, the development of RDTs is challenging, since RDTs are often low in sensitivity and specificity when compared to traditional, more time-consuming diagnostic methods, like the ELISA and plaque reduction neutralization tests (PRNT), which normally need hours to days to perform. 

Here we present an immuno-magnetic assay (IMA) for diagnosis of ZIKV infection based on the aggregation of magnetic nanoparticles (MNPs). The assay technology is implemented in a microfluidic cartridge format, which is designated ViroTrack Sero Zika IgG/IgM. The same technology has been implemented in detecting dengue NS1 and IgG/IgM as ViroTrack Acute Dengue NS1 Ag (cat. no. 01VTA02-25, BluSense Diagnostics, Denmark) and ViroTrack Sero Dengue IgG/IgM Ab (cat. no. 01VTS01-25, BluSense Diagnostics, Copenhagen, Denmark), which are commercially available products. The cartridge is used in combination with an opto-magnetic reader called BluBox (BluSense Diagnostics, Copenhagen, Denmark), which is designed for use in laboratories and decentralized settings. BluBox enables the quick diagnosis of multiple viral infections in a few minutes and requires minimal user interaction. The assay technology is based on the use of MNPs coated with viral antigens (ZIKV NS1) that react with ZIKV NS1-specific antibodies. These coated immuno-MNPs are deployed in a magnetic agglutination assay (IMA) with the samples. After incubation in a strong magnetic field, the target antibodies aggregate with immuno-MNPs, which are subsequently measured using an optical-based detection system in the BluBox reader. The details of the process are described on the website of BluSense Diagnositcs (https://blusense-diagnostics.com/technology/). IMA enables a novel, rapid and semi-quantitative ZIKV serological diagnostic method. Here, we present the evaluation of detection of ZIKV NS1-specific antibodies by the ViroTrack Sero Zika IgG/IgM cartridge and its performance in distinguishing between the ZIKV and DENV infections.

## 2. Materials and Methods

### 2.1. Ethics Statement

All patients’ samples were collected from different sources as indicated below and these serum experimentations were under the approval of the Research Ethics Board of Chang Gung Memorial Hospital in Taiwan (IRB 201601701B0). The Nicaraguan Pediatric Dengue Cohort Study was approved by the Institutional Review Boards of the University of California, Berkeley, and the Nicaraguan Ministry of Health. Parents or legal guardians of the subjects provided written informed consent, and participants 6 years of age and older provided assent.

### 2.2. ViroTrack Sero Zika IgG/IgM Cartridge Design and BluBox

**Figure 1:**
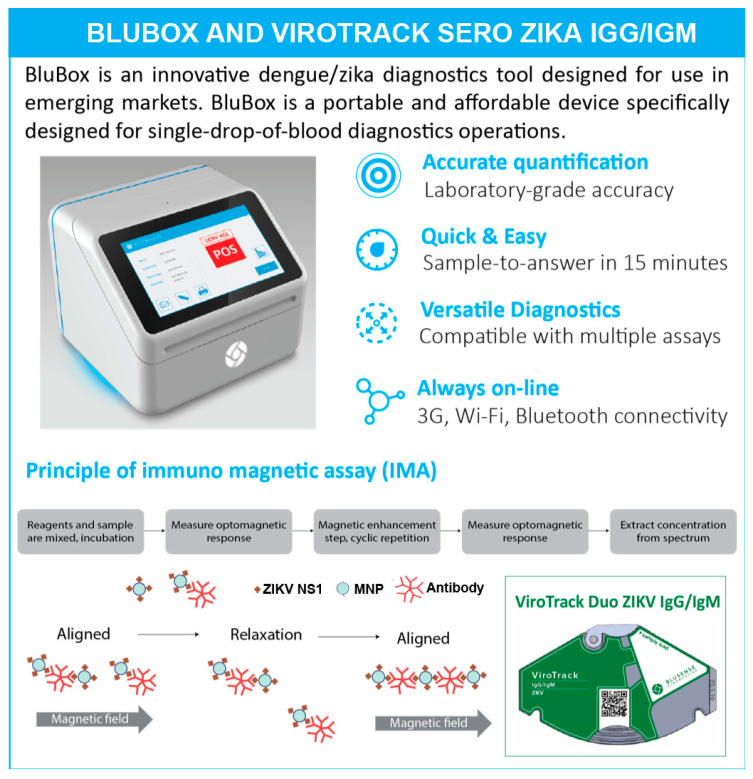
Blubox and ViroTrack Sero Zika IgG/IgM.

ViroTrack Sero Zika IgG/IgM—prototype and research use only version (BluSense Diagnostics, Copenhagen, Denmark) is a microfluidics-based semi-quantitative Zika detection assay based on IMA technology. It is designed as single-use and it is intended to be used in combination with the BluBox. ViroTrack Sero Zika IgG/IgM cartridge and the BluBox are illustrated in Figure 1. Each ViroTrack Sero Zika IgG/IgM cartridge has two detection pools, one for IgG and the other for IgM detection. The principle of IMA technology has been reported previously [33]. Briefly, the MNPs are coated with ZIKV-NS1 through “click” chemistry and immobilized on the cartridge. The ViroTrack Sero Zika IgG/IgM cartridge was loaded with 10 µL of diluted sample for each test, and the cartridge was then inserted into the BluBox. The sample sequentially dissolves the dry reagents, and ultimately the MNPs, in the detection chamber. In a Zika-positive sample, ZIKV-specific antibodies agglutinate the MNPs, which are detected by the readout unit. The level of the IgG or IgM antibodies in the sample is quantified in relative units (IMA units) by the BluBox. The results were interpreted based on a pre-set threshold value in the BluBox. The entire process is automated after loading the sample into the cartridge and takes less than 15 min to obtain both IgG and IgM results.

### 2.3. Clinical Samples and Sample Preparation

In this study, we included samples from different sources. Information about the samples is listed in Table S1 and S2 in detail. Samples from forty confirmed Zika cases from the Dominican Republic were purchased from Boca Biolistics, Pompano Beach, FL, USA. Thirty-six ZIKV-infected samples from the USA were provided by Biomedical Advanced Research and Development Authority (BARDA), Washington, DC, USA. One hundred and forty samples from laboratory-confirmed DENV- or ZIKV-infected patients were obtained from University of California, Berkeley, USA, which include six groups of samples from collaborative studies with the Ministry of Health and Sustainable Sciences Institute in Managua, Nicaragua. The pZIKV (Primary Zika) sample group is composed of primary ZIKV infected patient samples without prior DENV infection; the pDENV (Primary Dengue) sample group is composed of primary DENV-infected patient samples without prior ZIKV infection; the DENVpZIKV (Secondary Flavi infection) sample group is a group of patient samples with ZIKV infection after a prior documented DENV infection; the sDENV (Secondary Dengue) sample group is a group of samples from patients with secondary DENV infection and no previous history of ZIKV infection. All of these samples were collected between 14 and 44 days after illness onset, i.e., the early convalescent phase. Each sample group contains 25 patient samples. In total, 100 samples were tested by ZIKV IgG and IgM assay. In addition, there are two groups of DENV-positive samples collected in late convalascence, 3–9 months post-onset (MPO) of illness. Twenty samples are from primary DENV infections (pDENV_>3 MPO), and 20 samples are from secondary DENV infections (sDENV_>3 MPO).

All patient samples were diluted in sample dilution buffer (provided by BluSense Diagnostics) at a 1:20 dilution.

### 2.4. Immune Status

The status of ZIKV- and DENV-specific IgM and IgG antibodies in samples in this study was determined by different reference methods, for example, commercial ELISA kits (tested either in-house or by the provider), the CDC MAC-ELISA, or the dengue inhibition ELISA (iELISA) [34]. In-house ELISA tests (e.g., the ZIKV NS1 Blockade-of-Binding (BOB) ELISA [35], and ZIKV IgG/IgM ELISA kits from DIA.PRO) were used for ZIKV-specific IgG and IgM detection.

### 2.5. Serological Study 1

In serological study 1, we included three serial ZIKV samples (cat.no.1043-TDS-0220, 1043-TDS-0230 and 1043-TDS-0238) from the Dominican Republic (Boca Biolistics, Pompano Beach, FL, USA). The serum samples were diluted 1:20 with sample dilution buffer and evaluated with ViroTrack Sero Zika IgG/IgM and commercial ELISA kit ZIKA virus IgG and ZIKA virus IgM (DIA.PRO Diagnostic BioProbes s.r.l, Milan, Italy) according to the manufacturer’s instructions.

### 2.6. Serological Study 2

In serological study 2, we included ten naïve samples and 127 RT-PCR-confirmed Zika cases. These ZIKV-infected samples included 41 samples from the Dominican Republic (Boca Biolistics, Pompano Beach, FL, USA), 50 samples from Nicaragua (kindly provided by Prof. Harris from University of California, Berkeley) and 36 samples from BARDA collaboration (USA). All the samples were evaluated with ViroTrack Sero Zika IgG/IgM and commercial ELISA (DIA PRO for ZIKV, SD for DENV and EUROIMMUN for CHIKV for samples from Dominican Republic), ZIKV NS1 BOB ELISA [35] (for samples from Nicaragua), or CDC MAC-ELISA (for samples from BARDA collaboration) as reference (details are shown in Table S2). Among these ZIKV-infected samples, samples from four pregnant women were included.

### 2.7. Serological Study 3

In serological study 3, we included six different groups of samples from Nicaragua (kindly provided by Prof. Harris from University of California, Berkeley): the first group included 25 pZIKV samples; the second group included 25 DENVpZIKV samples; the third group included 25 pDENV samples; the fourth group included 25 sDENV samples; the fifth group included 20 pDENV_>3 MPO samples; the sixth group included 20 sDENV_>3 MPO samples (details are shown in Table S1). All 140 samples were evaluated with ViroTrack Sero Zika IgG/IgM according to the manufacturer’s instructions.

### 2.8. Statistical Analysis

In all violin plots, measurement data (points) with median value (center line), mean value (cross marker), minimum, first quartile, median, third quartile and maximum are presented. Zika IgG/IgM levels measured in different patient cohorts were compared using an unpaired t-test with confidence intervals of 98%. Results were considered to be significant at *p* < 0.02. ROC curve, area under curve (AUC), and optimized cutoff were carried out with R software.

## 3. Results

Evaluation of ViroTrack Sero Zika IgG/IgM for detection of ZIKV-specific IgG and IgM was performed in three sample sets. Serological study 1 shows serological evidence of ViroTrack Sero Zika IgG/IgM by testing ZIKV-specific IgG and IgM in samples collected from three ZIKV-infected patients at different days post-illness onset. In serological study 2, the sensitivity and specificity of the ViroTrack Sero Zika IgG/IgM cartridge was compared to standard techniques in a group of 127 ZIKV-infected samples and 10 healthy control samples. In serological study 3, four groups of samples—specifically, samples from primary ZIKV infections with no prior DENV infection (pZIKV); primary ZIKV infections with documented prior DENV infection (DENVpZIKV); primary DENV infections (pDENV); and secondary DENV infections (sDENV) in early convalescence were tested with ViroTrack Sero Zika IgG/IgM with the aim of assessing the cartridge specificity in relation to DENV infection.

### 3.1. Serological Study 1: Zika IgM & IgG Measured by VirTrack Sero Zika IgG/IgM Assay Following Three Patients after Illness Onset

**Figure 2:**
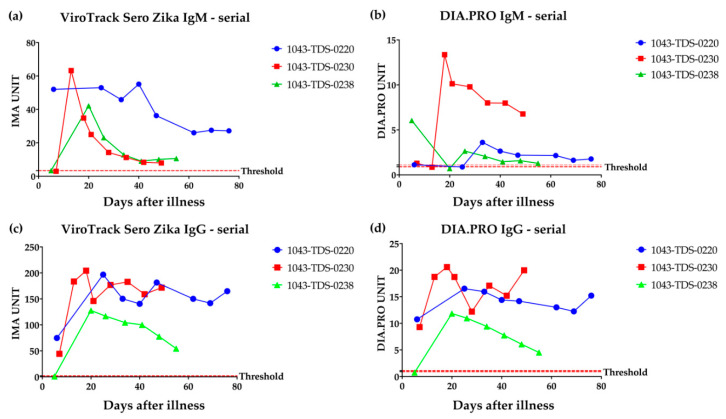
Serological study 1: ViroTrack Sero Zika IgG/IgM serological evidence study. (**a**) ZIKV IgM measured by ViroTrack Sero Zika IgG/IgM. (**b**) ZIKV IgM measured by DIA.PRO ZIKV ELISA kit. (**c**) ZIKV IgG measured by ViroTrack Sero Zika IgG/IgM. (**d**) ZIKV IgG measured by DIA.PRO ZIKV ELISA kit. ZIKV IgM and IgG values were measured by ViroTrack Sero Zika IgG/IgM for serial draw samples collected on different days from illness for 3 different ZIKV RT-PCR positive patients from the Dominican Republic. The expected seroconversion pattern is clearly measurable, and it is comparable with the ZIKV IgG and IgM patterns measured by commercial ZIKV ELISA kits.

ZIKV IgM and IgG values were measured by ViroTrack Sero Zika IgG/IgM (Figure 2a,c) in serial blood samples collected on different days post-illness onset (samples collected 5–76 days after symptom onset) for 3 ZIKV RT-PCR-positive patients from the Dominican Republic. These samples were also evaluated with the commercial ELISA kits, ZIKA virus IgG and ZIKA virus IgM (DIA.PRO Diagnostic BioProbes s.r.l, Milan, Italy) (Figure 2b,d). The expected seroconversion pattern is clearly measurable by the ViroTrack Sero Zika IgG/IgM, and it is comparable to the ZIKV IgG and IgM patterns measured by commercial ZIKA ELISA kits. One notable difference between the assays is the location of the IgM peak, which is observed at an earlier blood draw using the ViroTrack Sero Zika IgG/IgM assay.

### 3.2. Serological Study 2: ViroTrack Sero Zika IgG/IgM Sensitivity and Specificity

**Figure 3:**
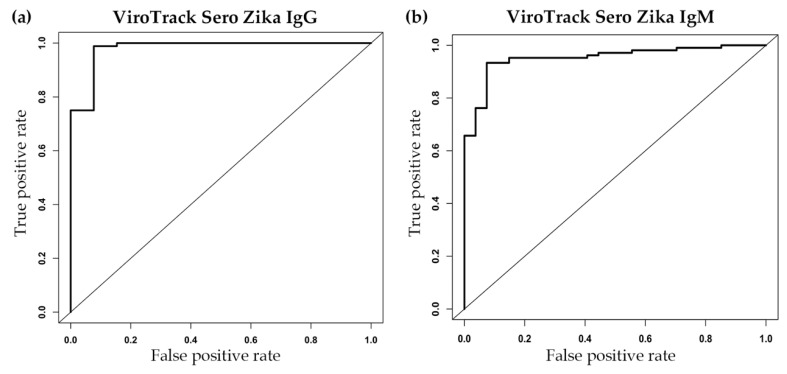
Serological study 2: ViroTrack Sero Zika IgG/IgM sensitivity and specificity. (**a**) ROC curve analysis by R for differentiation of 91 ZIKV-infected samples from 10 healthy control samples, based on ViroTrack Zika IgG measurement; and (**b**) ROC curve analysis by R for differentiation of 122 ZIKV-infected samples from 10 healthy control samples, based on ViroTrack Zika IgM measurement.

Promising results in terms of sensitivity and specificity for ZIKV IgG and IgM were obtained (Figure 3 and Table 1). Clear separation of the ZIKV-infected group from the healthy control group is observed—both ViroTrack Zika IgG and IgM yielded an AUC higher than 0.95 (see Figure 3a,b). The calculated sensitivity and specificity for ViroTrack Zika IgG is approximately 92% (both sensitivity and specificity), and for ViroTrack Zika IgM is around 93% (both sensitivity and specificity). The ViroTrack Sero Zika IgG/IgM results also show a high agreement with the reference ELISA results. The agreement for ZIKV IgG measurement is around 98%, and agreement for ZIKV IgM measurement is approximately 93%. The agreement for positive and negative samples is shown in Table 1.

### 3.3. Serological Study 3: ViroTrack Sero Zika IgG/IgM to Distinguish ZIKV- and DENV-Infected Samples

**Figure 4:**
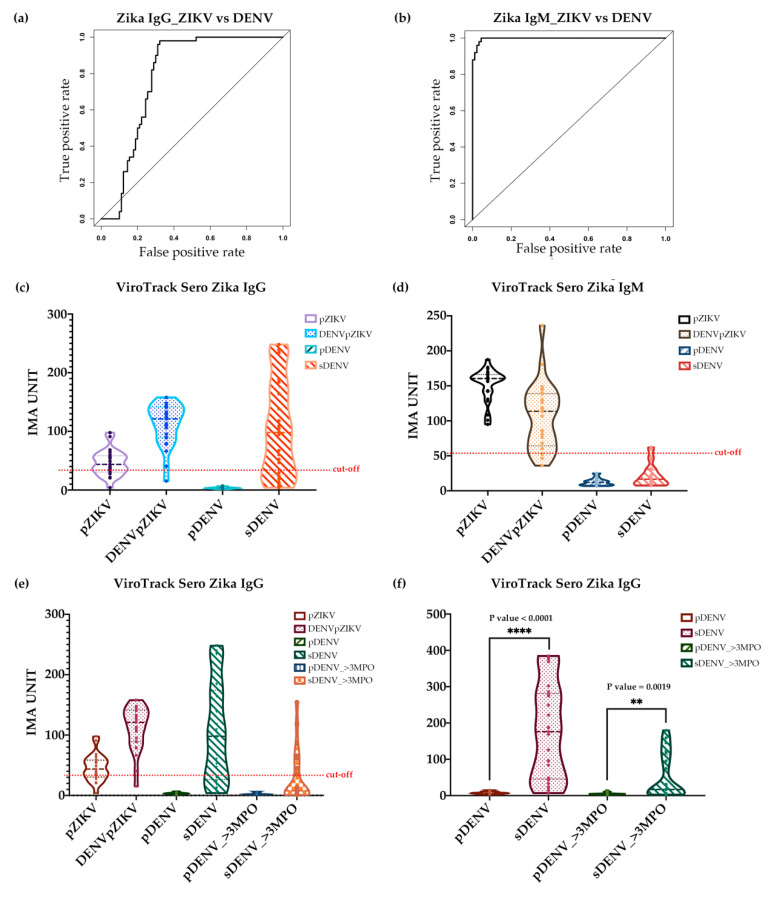
Serological study 3: Zika and dengue differential study. (**a**) ROC curve analysis by R for differentiation of ZIKV- from DENV-infected samples based on ZIKV IgG measurements: four groups of samples were analyzed, they are 25 pZIKV-infected samples, 25 DENVpZIKV-infected samples, 25 pDENV-infected samples and 25 sDENV samples; and (**b**) ROC curve analysis by R for differentiation of ZIKV- from DENV-infected samples based on ZIKV IgM measurements: four groups of samples were analyzed, they are 25 pZIKV-infected samples, 25 DENVpZIKV-infected samples, 25 pDENV-infected samples and 25 sDENV samples. (**c**) Violin plot of ViroTrack Sero Zika IgG/IgM measurements on ZIKV IgG and (**d**) IgM level from serum samples from four groups of samples as described in (**a**). (**e**) Violin plot of ViroTrack Sero Zika IgG/IgM measurements on ZIKV IgG level to compare primary and secondary DENV-infected samples from 25 serum samples with primary DENV infection collected at convalescent phase (around 20 days post-onset of illness), 25 serum samples with secondary infection collected at convalescent phase (around 20 days post-onset of illness), 20 serum samples with primary DENV infection (pDENV_>3MPO, sample collected 3 months post-onset of illness) and 20 serum samples with secondary DENV infection (sDENV_>3MPO, sample collected 3 months post-onset of illness). (**f**) Both sDENV samples from different stages of illness onset show significantly higher IgG levels than both groups of pDENV samples. ** *p* ≤ 0.05, **** *p* ≤ 0.0001.

The ability of the ZIKV assay to distinguish between DENV and ZIKV cases was investigated on four groups of samples from Nicaragua (kindly provided by Prof. Harris from University of California, Berkeley). In total, 140 samples were tested with ZIKV IgG and IgM assays, respectively. The optimized cutoff for the ZIKV IgG test is 32.17 IMA units, and for the ZIKV IgM is 51.92 IMA units. Results are shown in Figure 4. Figure 4a,b show the ROC curve analysis on both ViroTrack Zika IgG and IgM, ZIKV- and DENV-infected samples can be distinguished at a 96% level with AUC at 0.96 by ViroTrack Zika IgM measurement, while more cross reactivity is observed by ViroTrack Zika IgG. For ViroTrack Zika IgG, AUC is only 0.69 (Table 2), much lower than AUC of ViroTrack Zika IgM. ViroTrack Sero Zika IgG/IgM’s sensitivity and specificity are calculated in Table 2. For ZIKV IgG, sensitivity is 86.0% and specificity is 62.0%. For ZIKV IgM, sensitivity and specificity are calculated to be 96%.

The violin plots in Figure 4c,d show the distribution of all four groups of samples on ZIKV IgG and IgM measurements, respectively. A cutoff line is also shown on the graph. As shown in Table 3, 76% of pZIKV, 96% of DENVpZIKV, 4% of pDENV, and 72% of sDENV in ZIKV IgG were positive, suggesting that ViroTrack Zika IgG can distinguish ZIKV from DENV infections with a sensitivity of 86% and a specificity of 62%. Most cross reactivity happens with sDENV-infected samples, not much on pDENV-infected samples. When testing with ZIKV IgM, 100% of pZIKV, 92% of DENVpZIKV, 0% of pDENV, and 8% of sDENV in the panel were positive, which suggests very low cross reactivity in ZIKV IgM measurement. As a result, the ViroTrack Sero Zika IgG/IgM only seems cross reactive with sDENV-infected samples—specifically, in 8% of cases.

Because all of these four sample groups were sampled at the convalescent-phase, which is high in antibody titer, it was investigated whether the IgG cross-reactivity to ViroTrack Zika IgG can still be observed after a longer convalescence period. Therefore, two groups of samples were also included in the test: 20 samples with primary DENV infection collected 3 months post-onset of illness (pDENV_>3 MPO), and 20 samples with secondary DENV infection and no previous history of ZIKV infection collected 3 months post-onset of illness (sDENV_>3 MPO). These samples were measured with ViroTrack Sero Zika IgG/IgM for both IgG and IgM, and the result is shown in Figure 4e. Samples collected at a later stage show even lower cross-reactivity with ZIKV-infected samples, especially sDENV_>3 MPO. When it is compared the ZIKV IgG titer level on ViroTrack Zika IgG assay, the sDENV group sample shows a significantly higher IgG level than the pDENV group sample with *p* value < 0.0001. Furthermore, this difference is also observed from samples collected 3 months post-onset of illness. sDENV_>3 MPO shows a significantly higher IgG level than the pDENV_>3 MPO group sample (*p* value = 0.0019) (Figure 4f).

## 4. Discussion

ViroTrack Sero Zika IgG/IgM is a new microfluidic immunomagnetic magnetic nanoparticle-based immunoassay for serology detection of ZIKV infection developed by BluSense Diagnostics Aps. ViroTrack Sero Zika IgG/IgM precisely monitors the seroconversion pattern of three different ZIKV PCR positive patients between 5- and 76-days post-onset of illness. The detected trend of ZIKV-specific IgG and IgM titers is comparable with the reference ELISA results from DIA.PRO. Furthermore, the sensitivity and specificity of ViroTrack Sero Zika IgG/IgM are calculated to be 92% for IgG and 93% for IgM, using ELISA as a standard reference.

Due to the highly conserved region in their structure between DENV and ZIKV envelope proteins, it is very difficult to distinguish between ZIKV and DENV based on IgG or IgM tests by using the traditional envelope protein-based assay. The CDC-recommended IgM MAC-ELISA also holds limitations [36]. ZIKV NS1 has been given a great focus due to its ability to distinguish DENV and ZIKV infections. Wen-Yang Tsai et.al. developed a three ELISA combined assay to distinguish ZIKV and DENV infections, and it takes hours [37]. In our presented study, the ViroTrack Sero Zika IgM assay can clearly distinguish the ZIKV infection (group of both ZIKV primary infected and ZIKV-positive with previously DENV-infected samples) from the DENV infection (group of both primary and secondary DENV-only infected samples) with a sensitivity and a specificity of 96%, when sampling is collected at the convalescent-phase. However, our ViroTrack Sero Zika IgG assay found high cross-reactivity between the ZIKV infection group and the secondary DENV infection group. The cross-reactivity between DENV and ZIKV detection may be explained by the high similarities in their antigenic structure. Previous studies have demonstrated that sera from both acute and convalescent dengue patients could bind and neutralize ZIKV [38,39]. A recent study also indicated that the breadth of cross-reactivity to other serotypes and ZIKV in secondary DENV infection is greater than in primary DENV infection, which corresponds with our presented results [40]. Taken together, despite the fact that the cross-reactivity between ZIKV and secondary DENV infections still need to be well considered and evaluated, the ViroTrack Sero Zika IgG/IgM assay developed a rapid diagnostic assay for detecting ZIKV infection and potentially improved the distinguishability between the DENV and ZIKV infections.

In the next step, ViroTrack Sero Zika IgG/IgM should be evaluated on a larger sample size and more locations of sample origins. Further expansion of the cross-reactivity study with other flaviviruses such as Japanese encephalitis virus (JEV), West Nile virus (WNV), yellow fever virus (YFV) and tick-borne encephalitis virus (TBEV) is needed to precisely assess the cartridge specificity.

## 5. Conclusions

This section is not mandatory, but can be added to the manuscript if the discussion is unusually long or complex.

## 6. Patents

This section is not mandatory, but may be added if there are patents resulting from the work reported in this manuscript.

## Figures and Tables

**Table 1:** Comparison of ViroTrack Sero Zika IgG/IgM with reference ELISA assay.

Samples *n* = 137	IgG ^a^	IgM ^b^
Sensitivity	92%	93%
Specificity	92%	93%
Cutoff	11.5	12.14
AUC	0.98	0.95
Agreement to ELISA (%)	98	93

^a^: 36 samples are not included (no reference assay test); ^b^: 5 borderline samples are not included.

**Table 2:** ZIKV IgG and IgM sensitivity, specificity and AUC are calculated respectively.

ViroTrack Sero Zika IgG/IgM	Sensitivity	Specificity	AUC
Zika IgG	86%	62%	0.69
Zika IgM	96%	96%	0.96

**Table 3:** Results of ViroTrack Sero Zika IgG/IgM in different sample groups: pZIKV, DENVpZIKV, pDENV and sDENV.

ViroTrack Sero Zika IgG/IgM	pZIKV	DENVpZIKV	pDENV	sDENV
Zika IgG	19/25 (76%)	24/25 (96%)	1/25 (4%)	18/25 (72%)
Zika IgM	25/25 (100%)	23/25 (92%)	0/25 (0%)	2/25 (8%)

## Supplementary Materials

Supplementary Materials can be found at https://www.mdpi.com/2075-4418/10/6/372/s1.

## Abbreviations

DENV	Dengue virus
ZIKV	Zika virus
CHIKV	Chikungunya virus
JEV	Japanese encephalitis virus
WNV	West Nile virus
YFV	Yellow fever virus
TBEV	Tick-borne Encephalitis virus
GBS	Guillain-Barré Syndrome
RDT	Rapid diagnostic test
PRNT	Plaque reduction neutralization test
ELISA	Enzyme-linked immunosorbent assay
BSD	BluSense Diagnostics
IMA	Immuno-Magnetic Assay
MNPs	Magnetic nanoparticles
BARDA	Biomedical Advanced Research and Development Authority
pZIKV	Primary Zika infection
pDENV	Primary Dengue infection
DENVpZIKV	Primary Zika with documented DENV infection
sDENV	Secondary Dengue infection
MPO	Months post-onset
iELISA	Inhibition ELISA
BOB-ELISA	Blockade-of-Binding ELISA
MAC-ELISA	IgM antibody capture enzyme-linked immunosorbent assay
ROC	Receiver operating characteristic
AUC	Area under curve
